# M. Piorry on Exploration of the Thoracic and Abdominal Organs, by Means of Percussion

**Published:** 1834-04-01

**Authors:** 


**Du Procédé Opératoire a suivre dans l'Exploration des Organes, par la Percussion Mediate, &c. Par *P. A. Piorry*, &c. &c. &c. Paris, 1831, in 8vo.**

Although percussion is now very commonly employed in this country in conjunction with auscultation in our examinations of the state of the thoracic organs, it is not generally—or rather we believe—it is very rarely performed in the systematic method recommended by M. Piorry. To those who are not fully aware of the extent to which such a mode of percussion may be made available in discovering various morbid conditions, in situations where auscultation alone has failed or been inapplicable, the analysis we are about to make of the work whose title is prefixed to this article will not, we persuade ourselves, be wholly unacceptable.

The object of percussion is to ascertain the condition of those organs which are within the reach of its influence. M. P. professes to obtain this object more or less completely by an appreciation of the various sounds elicited and sensations caused by percussion over the parts examined through the medium of a thin circular plate of ivory, box-wood, copper, &c.

Whatever adds precision to diagnosis, however apparently insignificant the observation, or simple the device, is valuable. Why interpose this remark? The generation has not yet passed away among whom Laennec's cylinder was—we might say is—the object of sneering and derision—that simple instrument, which his genius, his ardour of research, and patience of observation, have rendered the means of effecting changes in a great division of pathology that the most sanguine could scarcely have anticipated. Should the eyes of any of that generation glance upon these pages, their contents, we would forewarn them, are addressed to readers of another class —to students who have really laid their hand to the plough of their profession—students who are earnestly engaged in the pursuit of truth, and ready to hail it whenever and whencesoever it may appear as light from Heaven.

Let those who, in obedience to a wise scepticism, doubt the extent of the utility which M. Piorry ascribes to his process, search for themselves upon the dead subject, and compare the state of parts with the sounds elicited from them by percussion. Let them compare the results obtained in the dead with those in the living subject. The liver—the spleen—the lungs—the intestinal canal distended with gas, must necessarily yield the same sounds immediately after death that they did immediately before. Death does not in an instant change the physical consistence of tissues: it is not life that makes them hard or soft—large or small—full or empty; a gas, a liquid, or a solid, whether they exist in the living or dead subject, must still possess the consistence of a gas, a liquid, or a solid. And the results of percussion therefore must, cæteris paribus, be similar before to what they would be after death.

The method latterly recommended by M. P. as facilitating the study of pleximetry, has been to practise on the different organs laid bare. The ear is thus made acquainted with the sounds yielded by the different tissues, and enabled to recognize them more readily afterwards in the living subject. The proceeding seems rational, and we doubt not will be adopted by those whose zeal corresponds with their opportunities. Let it not be supposed, however, that two or three trials are sufficient to enable the experimenter to decide upon the merits of our subject. Our senses require education; and it is of very recent date that we have begun to cultivate that of hearing—at least in reference to the peculiar class of sounds, the appreciation of which has been rendered so important by the application of auscultation and pleximetry to pathological investigations. Nor is the ear the only organ through which M. P.'s process furnishes us with information. The success of our pleximetric examinations will very much depend on our readiness in appreciating the sensation produced in the fingers with which the operation is performed. It is by means of this sensation for instance that we are able to infer at once and very exactly not merely the density of integuments, but that of the liver covered by them, and separated from them by the intervention of ribs and a portion of lung distended with air. To do this however on all occasions, and, as we have said, very exactly, requires practice. "Pleximetry," observes M. Piorry, "constitutes a part of the chirurgy of diagnosis: and the knowledge necessary to the performance of the simplest operation is not to be acquired without study."

In imitation of Laennec, our author has assigned names to particular sounds, but has not, in our opinion, been very happy in his nomenclature. To the sound produced by percussion on the frontal bone, where the finger experiences the maximum of resistance, he has affixed the term *osteal.* The terms jecoral, cardial, stomachal, &c. indicate the parts whose sounds and impressions he has adopted as types. Percussion on the fleshy part of the thigh produces a double impression of hearing and touch representing the sound and density of the left ventricle. The parts of the liver in contact with the ribs yield a duller sound and offer more resistance than the thigh.

The type of the sound and elasticity of the lung is perceived by percussion on the chest at the central point between the nipple and clavicle. The *most* perfect type of what is called *humoric resonance* is obtained by placing the palms of the hands together, so as to enclose a certain portion of air, and then as children do to produce the sound of money—striking the back of one of the hands on the knee.

This humoric or metallic resonance indicates air escaping by a narrow opening from a spacious cavity: the term *humoric* is therefore manifestly ill-chosen—since it implies that the production of the sound in question is connected with a liquid, which is not necessarily the case.

The type of the sensation produced by percussion over a large cyst of hydatids is nearly approached by tapping jelly of firm consistence—or it is still more exactly represented, according to M. Piorry, in the feeling of vibration perceived if we place a repeating watch on its back in the palm of the hand. He deduces from certain experiments that the vibration is perceptible in proportion to the quantity of the acephalocysts relatively to the fluid in which they are immersed.

To proceed however to the author's directions for studying pleximetry in the dead subject.

"The anterior parietes of the thorax and abdomen being removed so as to leave the parts beneath as nearly as possible in their natural position, percussion should be practised on each part in situ. The clear sound of the lung and the dull sounds peculiar to the heart and liver will be heard:—The differences of resistance and of resonance furnished by the right side of the heart gorged with fluid, and the more muscular left side containing much less blood. The dull sound caused by the portion of liver, which pressing the diaphragm upwards, rises under the inferior margin of the right lung a thin layer of which is spread over it.

The resonance produced by percussion of the liver will vary from above downwards: above, where it is thickest, the sound will be most obscure, and the resistance greatest: below, with the sound peculiar to the liver, there will be joined the tympanitic sound caused by the presence of the intestinal canal filled with gas. On the point corresponding with the gall-bladder will be found sometimes dull sound without resistance—at others the humoric resonance. (?) The stomach—cæcum—large intestines—if much distended with gas, will yield their peculiar tympanitic sounds—if they contain liquids, the humoric resonance, whilst they offer no resistance to the finger with which percussion is made.

A body will next be opened from behind. Here the lungs will generally be found to yield a more obscure sound, and to present rather more resistance to the finger than anteriorly, in consequence of cadaveric engorgement. Below these organs the sound and resistance peculiar to the liver will be remarked on the right side, and on the left the dull sound and resistance caused by the spleen, combined with the elasticity of the neighbouring portions of the digestive tube:—lower down a duller sound and impression of greater density will be furnished by the kidneys—and external to these the tympanitic resonance of the large intestines filled with gas will be obtained."

"The bladder may be examined by injecting water into it, in different quantities, when it will afford its corresponding impressions both to the ear and touch;" and, in like manner, the physical conditions of the other organs may be varied for the purposes of experimental examination, *e.g.* by forcing air into the lungs their elasticity and sonoreity may be increased—or the reverse, by injecting them with water. The right side of the heart may be dilated by injecting it from the vena cava—and sounds and degrees of resistance, corresponding with the changes thus produced, obtained. Artificial effusions in the pleura—pericardium—peritoneum—may also be advantageously studied. In all cases in which the physical conditions of tissues are changed by disease, this method of examining by percussion, after having exposed them, is recommended, until a knowledge of the sounds and impressions of density peculiar to each is attained. This being done, we shall be capable of appreciating those produced by similar conditions in the living subject.

Let us now accompany M. Piorry to the pleximetric examination of particular organs or parts—and firstly,

### Of the Lungs and Pleura.

We would previously make the general remark, that the muscles, on which the pleximeter is applied, should be as relaxed as possible. It is especially important to remember this in examining the abdomen; for a contracted muscle will afford a sensation of hardness, due merely to its contraction, and of course not present when it is relaxed. It is not necessary to apply the pleximeter to the skin, but the lighter and fewer the materials interposed the better. The pleximeter should be held firmly between the thumb and fore-finger, and applied very exactly to the surface, so that no air may escape from any part of its circumference, whence would result the metallic resonance already alluded to.

"The person to be examined may be either in a sitting or recumbent posture. The pleximeter should be placed at the head of the sternum; the strokes upon it should be first gentle, then stronger; the sound in this position will be generally tympanitic, and the impression conveyed through the finger, that afforded by an elastic body. The instrument will be brought gradually downwards along the median line until the sound becomes obscure, and a certain degree of resistance is perceived (this will, in most cases, be about three inches above the xyphoid cartilage; it is caused by the right side of the heart—is not very perceptible *to a beginner*, and requires pretty strong percussion to be rendered sensible.) The spot where this change is remarked should be carefully noted. The lateral regions should be examined with equal care. To examine the triangular space above the clavicle, and which corresponds with the summit of the lung, the head should be inclined to the side opposite that examined. Below the clavicle, percussion, as before, should be performed from above downwards, and in succession over the whole surface of the chest. To judge of the degree of elasticity or density of the lungs, the patient should be directed to make as full an inspiration as he can, percussion being made at the same time; then a forced expiration, when percussion is again to be performed, and the results in these two conditions compared. If the lung be indurated, there will be no more elasticity or sound at the diseased part in the one case than in the other; whilst, in a nearly healthy lung, the difference will be very sensible."

In very emaciated subjects, notwithstanding M. Piorry's ingenious contrivances to adapt his pleximeter to the unequal surface caused by the projecting ribs, we beg leave, with great deference, to observe that the hand is by far the better instrument. If the integuments are very thick, from obesity, emphysema, or œdema, the pleximeter should be pressed firmly down upon them, so as to rest as nearly as possible on the ribs. The same observation applies to the mamma—or, when large, it should be pressed to one side.

Percussion should be made alternately on corresponding parts of each side of the chest; first gently, to ascertain the condition of the superficial layers of the lung—then more strongly, to discover the state of its more deeply-seated portions. At the lower part of the right lung, as the instrument is carried downwards, the sound becomes gradually more obscure, and the sense of resistance greater, until, at length, we obtain the pleximetric signs denoting the presence of the liver. In a healthy subject, the sound becomes dull, and the resistance is perceived higher up on the left side than on the right. The presence of the heart is indicated, extending sometimes as far as the margin of the ribs. The situation and extent of this change of sound and density vary, according as the stomach is full or empty, the spleen hypertrophied or otherwise, &c.

We cannot follow M. Piorry through his minute directions for examining the posterior surface of the thorax—suffice it to observe, that percussion should be performed over every part that covers a portion of lung.

*Pleura.* When pleuritic effusion is suspected, the examination should be commenced posteriorly; if it exist, there will be a dull sound on percussion at the most dependent part of the thorax—at the lowest part of the right side there will, in the normal state, be always a dull sound, caused by the presence of the liver : but, if there be no effusion, a gentle percussion will elicit a certain degree of sonoreity, sufficient to indicate, to a practised ear, the presence of the thin layer of lung that intervenes between the superior surface of the hepatic organ and the ribs.

M. P. says that the observation should be extended to left side, especially if the spleen be enlarged.

When the dull sound is observed above the superior margin of the liver on one side, or of the spleen on the other, its limits should be marked with nitrate of silver; we are next to examine the lateral and anterior parts of the thorax, noting the parts whose sonoreity and elasticity are most perfect; the position of the patient being then changed, so that the fluid, if there be any, may gravitate into those parts. If the part where the dull sound previously existed have recovered its sonoreity, and the sonorous parts have become dull, we may be satisfied of the existence of effusion. To be certain of it, we may repeat the process as often as we find it necessary, and, as M. Piorry observes, with a jealous regard for the honor of the process, "we should never give an opinion as long as a doubt hangs over our mind."

There are certain morbid conditions which it will be well to remember, in order to guard against errors into which we might otherwise be led.

1. An effusion may be limited by surrounding adhesions to a particular spot, and that not the most depending. If, within this space, the fluid has room to move from one part to another, percussion, by eliciting now a dull, and now a sonorous sound, according to the position of the patient, will reveal the morbid condition. This partial displacement, however, it must be confessed, rarely happens, and then there is often no mode of ascertaining whether the dull sound proceeds from effusion, pneumonia, induration of the lung, &c.

2. An adhesion may retain the lung at a particular point of the thoracic parietes, whilst the rest of the pleura is filled with serum. At that point, the sonoreity will be observed and respiration heard.

In cases of extensive effusion, we shall still find above the clavicle the clear sound and elasticity of the lung. In such cases, also, it may be observed, the dull sound may extend beyond the median line into the healthy side, the heart may be more or less displaced; and when the effusion occurs in the right pleura, the liver may be forced several inches below the margin of the ribs. All these circumstances should enter into the formation of our diagnosis. In such cases, moreover, mensuration of the thorax and the convex appearance of the intercostal spaces, greater on one side than on the other, will also contribute to its certainty.

*Lungs.* M. Piorry occupies several pages in showing that the art of diagnosis, in parenchymatous inflammation of the lungs, is far from having arrived at the high degree of perfection that many suppose. He observes that, in numerous instances among the aged patients of the Salpetrière, the *prune juice* expectoration has occurred where there was no pneumonia—where there was no other disease than obstruction of the circulation of the heart; on the other hand, that the most intense pneumonia has existed, unaccompanied by such expectoration, with very little embarrassment of respiration, and without pain of side; and this he declares himself to have witnessed in hundreds of cases!

"The crepitous rattle being nothing more (he observes) than sounds produced by air passing through liquids effused in the minutest bronchial ramifications, almost where they terminate in the air-cells, is not more diagnostic of pneumonia than of some other diseases of the lungs, in which an analogous condition exists, serum, blood, or any liquid, poured out into those very minute branches of the bronchial tree, will serve for its production. It is more frequently heard in pneumonia than in other pulmonary affections, because it is the nature of that disease that it should exist amongst, and, in its course, cause effusion of fluid into, the minutest bronchial ramifications. But the crepitous rattle occurs also in other diseases, as asphyxia, *par l'ecume bronchique*—pulmonary œdema: it is also met with in the cases of the aged, whose debilitated organs are not equal to the task of disencumbering the lungs of the fluids they contain."

This sign, however, we are happy to find, when it occurs in connexion with those furnished by mediate percussion, perfectly satisfies M. Piorry; so it does us.

We think our author might have saved some valuable time, had he merely stated generally—that, when we have several means of investigation, we should not be satisfied with the signs furnished by only a few of them—at least in any case of a doubtful character. He adduces, as an illustration of this, the circumstance, that pectoriloquy may occur with mere induration of a portion of lung, a fact denied by Laennec, but which appears to have been established by Cruveilhier; and the author declares that he has heard the voice passing up the tube from a part where there were only a mass of tubercles, or a portion of hepatized lung permeated by a large bronchial tube. On the other hand (the experiment is worth recording), he has ascertained that, on auscultating the larger bronchi in a dead subject, whilst another person spoke into the larynx, or a cavity attached to the trachea, representing the larynx, if the utterance was distinct, the most perfect pectoriloquy was obtained. If the voice was particularly strong and sonorous, there was bronchophony; if weak and sharp, œgophony. On coughing or breathing into such a cavity, the tubal voice (voix tubaire) and cavernous respiration were heard; and certainly there appears no reason why similar results should not be obtained in the living subject.

With regard to the signs obtained by mediate percussion, in disease of the pulmonary parenchyma, they may be briefly stated.

Pulmonary congestion is not distinguishable, by percussion, from the first stage of pneumonia: in both, the sound is more obscure than when the lung is healthy, and the resistance to the finger greater. In the second and third stages of pneumonia, the obscurity of sound and resistance are considerably greater.

When there exist extensive tubercular masses, the sense of resistance is yet greater, in some cases almost approaching to that of bone. If induration exist only in the centre of the lung, a strong percussion will be attended by a sense of resistance and an obscure sound, proportioned to the depth of the physical lesion.

Metallic tinkling has almost invariably indicated tubercular cavities: to obtain it, percussion should be made with a certain degree of force, and the finger quickly raised. The size of the cavity may be accurately measured, by marking the points around it where the dull sound commences.

A singular case is narrated, of a portion of lung forming a tumor at the posterior and inferior part of the thorax, near the spine. It was recent, of about a hand's breadth, and projected about an inch and a half above the surrounding surface. Some fancied they perceived an obscure fluctation in it, and it was on the point of being opened, when M. Michow, by whom the case was communicated to M. Piorry, obtained by mediate percussion a clear sound from the swelling. It was at once evident that the tumor contained either a fold of intestine or a portion of lung. On auscultating, the respiratory murmur was heard, and the real nature of the case of course made clear. M. Piorry remarks that, "here, direct percussion would have been impossible." Why? The Doctors thought it advisable to leave the tumor to itself. Not so the patient; she applied to a midwife, who, with the unpremeditating courage of ignorance, plunged a bistoury into it. A little matter flowed out—a portion of lung was seen through the wound, and the two last ribs partially destroyed by caries, in consequence of which the lung had been enabled to escape.

We next proceed to the consideration of mediate percussion, as applied to the exploration of the heart and its appendages. Here, we must confess, our author's certainly not unnatural admiration of a process, whose utility he has so ably developed, makes him somewhat prolix. It is unnecessary to occupy much space in shewing that auscultation alone, although it may, and in very many cases does, most materially assist in the formation of correct diagnoses, is much less useful as applied to diseases of the heart than those of the lungs. M. P. cites many cases in proof of this; some in which there existed a distinct and unintermitting bellows-sound and bruit de râpe in the region of the heart, which led him to expect ossification and contraction of the auriculo-ventricular or arterial orifices; but dissection disclosed only hypertrophy of the organ. Dr. Elliotson has offered an explanation of this circumstance, namely, that "the bellows-sound will be heard whenever the *relative* capacities of the orifice and ventricle are altered." The physical cause of the sound is the increased friction of the blood against the sides of the ventricle or vessel through which it is passing. This explanation accords with the results of the more recent researches of M. Bouillaud, published in one of the Numbers of the Journal Hebdomadaire for June last. According to this accurate observer, the increase of friction, to which the bellows-sound is to be attributed, may occur principally in three conditions—to wit, 1, when the motive powers of the heart are increased; 2, when there is contraction of any part of the canal through which the column of blood has to pass; and, lastly, when there is any lesion which renders the inner and naturally highly-polished surface of this canal rough or jagged.

In examining the heart, we should have an accurate recollection of the relative situation of parts—the relations of the heart with the pleura, the right lung, the liver, and the stomach, both covered by the diaphragm. It should be remembered that the space occupied by the heart is, posteriorly, at a distance from the thoracic parietes, from which it is also separated by a layer of lung, of considerable thickness—that, to the left, it is much nearer the parietes—that, anteriorly, it touches them—that the left auricle, situated at its postero-superior part, is covered by a layer of lung—that the heart's position is oblique, so that its greatest diameter is from right to left, near to where it rests on the diaphragm, its dimension from above downward being less.

As has been already recommended, it will be useful, where there is the opportunity, to inject the cavities in succession, in order to study the pleximetric signs of the condition so represented. The pericardium also may be injected, either with fluid or air, to observe its changes of position, having previously to these experiments tied the trachea, to prevent the lungs collapsing when the thorax is opened.

We are directed to carry "the pleximeter from above downwards along the median line until the sound begins to be dull; the spot where this occurs is to be marked with nitrate of silver; percussion is then to be made to the right, to ascertain the situation of the upper margin of the liver, which is to be carefully defined, the pleximeter being gradually carried along it towards the left, until a deeply-seated obscure sound, not attended by resistance to the finger, is observed: this spot, after it has been distinctly ascertained by repeated trials is to be marked with caustic. The instrument will then be carried on, and generally a marked obscurity of sound, and resistance to the finger will be observed; this indicates the left ventricle, and will be distinct or not according to the thickness of its parietes, the solidity of its tissue, and its vicinity to the thoracic parietes; farther to the left the sonoreity and elasticity will gradually return."

We are thus enabled to trace with the utmost degree of precision, every point of the right and left superior portions of the heart's circumference, its relative position to the lungs, the point where the right ventricle terminates and the left begins. If the right side of the heart, however, be much distended with blood, it will cover the left side so as to prevent our ascertaining its physical condition.

In tracing its inferior circumference the principal difficulty is caused by the vicinity of the liver; the limits of this viscus are to be ascertained, when, except in some rare cases, we shall easily be enabled to mark the situation of this part of the heart.

The following alterations of situation and sound will be observed in connexion with the morbid conditions detailed below.

1. In extensive effusion into the pericardium, the dull sound is found extending longitudinally, and to the upper part of the sternum, rather than from side to side.

2. In dilatation of the right ventricle the organ extends above the upper margin of the liver, and to the right of the median line. M. Piorry declares that he has known the space thus occupied, increase and become enormous during a paroxysm of dyspnœa, almost amounting to asphyxia, and in a few instants after the paroxysm was over, and the respiration had resumed its normal state, the extent of the space was reduced to nearly its natural proportions; the sensations furnished by percussion in this state are precisely the same as those afforded by the pericardium filled with water.

3. Dilatation of the left ventricle yields a remarkable dulness of sound to the left of the cardiac region over a space proportioned to the extent of the disease, sometimes five, six, or even seven inches. If hypertrophy exist at the same time, the resistance to the finger will be proportionally great. If hypertrophy uncomplicated with dilatation, the space occupied by the left ventricle rarely exceeds its natural dimensions, but the resistance to the finger will be very great.

4. Dilatation of the right auricle is indicated by a dull sound with little resistance at the superior part of the right cardiac region. M. P. has not observed in the living subject the signs furnished by dilatation of the left auricle.

All the cavities of the heart, but more particularly those of its right side, vary considerably in volume, according to the quantity of blood in the subject, to obstacles to the pulmonary circulation, or as there are contractions of the cardiac orifices. The heart's volume often diminishes very remarkably after bloodletting, sometimes to the extent of several inches: this may be demonstrated by pleximetry, marking with caustic the space occupied by the organ before and after venesection. These changes of volume are valuable diagnostic signs, as indicating dilatation rather than hypertrophy.

Hydrothorax on the left side, combined with disease of the heart, may be ascertained by pleximetric examination, by changing the patient's position so that the fluid may gravitate, as already observed when considering pleurisy.

Tubercles, when they exist to a considerable extent in the portion of the lung surrounding the heart, will prevent the possibility of tracing its circumference.

The stomach, intestines distended with gas or food, ascites, a tumor, hypertrophy of the spleen, or of the small lobe of the liver, may force the heart from its natural situation into those noticed above, as occupied by it in disease of its own tissue; by a careful examination however the cause of the displacement should be disclosed.

To examine the abdomen its muscles should be relaxed by having the legs gently bent, and the head somewhat elevated by pillows. The pleximeter should be pressed firmly on the integuments, especially if they are thick or œdematous.

Percussion being made along the median line from sternum to pubes superiorly there will be found the dull sound and resistance peculiar to the liver; lower down the tympanitic sound of the stomach and intestinal canal, varying according to the capacity of the different organs and the quantity and nature of their contents. Still lower, if the bladder be distended with urine, there will be found a dull sound, and slight resistance to the finger, yielding a kind of pasty feel, which it is scarcely possible to describe, and requires some practice to recognize.

Percussion should in succession be made over the whole surface of the abdomen, at first gently to ascertain the condition of its superficial contents, then more strongly to examine those which are more deeply seated.

In *peritoneal effusion* of no great extent, the parts around the umbilicus will be more sonorous than usual, on account of the fluid situated in the most dependent parts of the abdomen forcing the intestinal gases into that region; from this point we should percuss downwards until the sound becomes obscure; mark carefully the limits of the situation in which this occurs, and afterwards changing the patient's position, allow the fluid, if there be any, to gravitate—in fact, precisely as has been already recommended in pleuritic effusion.

When the effusion distends the abdominal parietes so that the intestines are no longer in contact with them, there will be dull sound on percussion every where; in this case fluctuation will be so evident that there could be no mistaking the disease.

We do not deem it necessary to follow M. Piorry into all his minute details, shewing how even a few ounces of serosity may be detected, when, in consequence of peritoneal adhesion, it is partially confined in a small space. Sufficient notice has been already taken of the subject, and M. P.'s farther directions will naturally suggest themselves to those who are studying the subject. In the mean time we would observe that it really is one worth studying, when we consider of how great importance it is to detect the existence of peritoneal disease in its commencement; how insidiously it often makes its approaches; and how obscure and liable to misinterpretation are many of its symptoms.

There are few, perhaps no, cases in which tenderness of abdomen will prevent the application of pleximetry if well performed: the instrument should be held firmly, and percussion performed very lightly with one finger. We should change the position of the patient slowly and cautiously, observing any unusual resonance of the intestines both superiorly and in the more dependent parts, and any change in the degrees of sonoreity subsequent to an altered position.

The nature of abdominal tumors may sometimes be conjectured by the sensation they produce in the finger on percussion, schirrus, encephaloid or fibrous tumors, not softened, impart a sensation of greater density and hardness than an abscess or cyst, whose walls are not very thick.

In examining the liver the stomach should be empty, the patient lying as for exploration of the abdomen; the part where the organ, through the medium of the diaphragm, is connected with the heart, should be carefully marked, as well as the margin of its superior surface below the lung, the extent to which this latter organ descends upon it should be carefully examined.

Percussion may first be made below the nipple, proceeding from above downwards, until the clear sound and elasticity indicate the presence of the stomach or intestines; then from below upwards to obtain the same results in an inverse order. At the middle of the organ percussion should be strong, lower down it should be gentle, to examine the thin layer of the viscus, which is there in contact with the parietes, and covers a portion of intestine. Percussion may then be made from right to left, observing where the left portion of the organ terminates. By marking these different points as we proceed with caustic, we shall have a very exact outline of the organ, and a practised experimenter will judge very accurately of the thickness of the liver by the degree of obscurity of sound, and the resistance it presents.

The height to which the liver rises in the thorax may be ascertained with the utmost precision, a circumstance of no little importance in certain cases. The inferior margin of the liver sometimes ascends several inches above the lower ribs; whilst its superior margin reaches above the nipple. If in such a case the organ be the seat of pain, the dull sound on percussion in such a situation might mislead a superficial observer, and induce him to suppose it connected with pneumonia, tubercular infiltration or pleuritic effusion, according to the nature of the concomitant symptoms. To avoid such an error we should seek the tympanitic sound of the intestine below the liver, and then carrying percussion upwards till we arrive at the viscus, we shall at once perceive that the digestive canal is pressed upwards under the ribs, whilst the liver ascends proportionally into the thorax. Having done this we ascertain its volume in the way already described, and we satisfy ourselves that there is no pleurisy or pneumonia by the means appropriate to the diagnosis of those diseases.

This displacement of the liver may occur in cases in which there is meteorism of no very great extent, and may be partly owing to the contraction of the abdominal muscles. In its normal condition the liver generally rises to one or two inches below the nipple, and its lower edge terminates at the margin of the ribs.

According to M. Piorry's pleximetric observations, it appears that the liver undergoes much more frequent and rapid changes of volume than is commonly supposed. He has known its volume to decrease after venesection to the extent of from one to three inches from above downwards in 24 hours; and this not only in the cases of old men whose venous circulation may have been obstructed by organic disease, or by mere debility of the heart, but in young persons labouring under plethora or attacked by acute fevers.

He relates a case of icterus in which the viscus increased in volume as the yellow hue of the integuments became more intense; as the latter faded, the liver decreased, the colour deepened, the size of the organ again increased. From these and analogous facts he arrives at certain physiological conclusions respecting some of the uses of the liver, which we can here only hint at. The sum of them is, that it serves as a reservoir of blood intended to regulate the state of the circulation. If there be too much blood in the system the organ augments—if too little its volume decreases. A similar function, but in an inferior degree, is attributed to the lungs and muscles.

Two very distinct conditions have been confounded under the common term hepatitis: one is mere congestion: in the other there exists some source of irritation in the organ, causing a determination of blood to it, and which is truly inflammatory. These congestions cause icterus, for the hepatic granulations being distended with blood, must press upon the biliary ducts and obstruct their circulation.

In slight hydrothorax the liver is easily examined, varying by change of position the situation of the pleuritic effusion. When it is considerable, the liver will be forced downwards, and in this case it may be impossible to ascertain its volume. We may be equally at a loss in cases of induration of the lower lobe of the right lung, which cannot be distinguished from the liver, unless there be a few bronchi still permeable.

In ascites as in pleuritis the situation of the fluid must, if necessary, be changed by varying the position, in order to trace the outline of the liver, and consequently to ascertain its dimensions.

Percussion, with the view of discovering the condition of the gall-bladder, we are disposed to regard as one of the refinements of the art, the consideration of which we may venture to postpone. M. Piorry declares that its situation may be recognized by the humoric resonance it affords on percussion. We are neither prepared nor inclined to dispute his authority; but it does not seem quite impossible that the neighbouring intestines may have some influence in the production of the phenomenon.

M. Piorry extends the application of pleximetry to examination of the spleen, stomach, intestines, kidneys, bladder, and womb. In the course of the preceding pages, we believe we have extracted the pith and marrow of the attributes of his *procédé*. The principles laid down for exploring the organs that we have been considering may be readily applied to those we pass over. We take leave of the subject with feelings of respect for our talented author, and earnestly commend his process to the attention of our professional brethren.

